# Quercetin Rejuvenates Sensitization of Colistin-Resistant *Escherichia coli* and *Klebsiella Pneumoniae* Clinical Isolates to Colistin

**DOI:** 10.3389/fchem.2021.795150

**Published:** 2021-11-25

**Authors:** Yishuai Lin, Ying Zhang, Shixing Liu, Dandan Ye, Liqiong Chen, Na Huang, Weiliang Zeng, Wenli Liao, Yizhou Zhan, Tieli Zhou, Jianming Cao

**Affiliations:** ^1^ School of Laboratory Medicine and Life Science, Wenzhou Medical University, Wenzhou, China; ^2^ Department of Clinical Laboratory, the First Affiliated Hospital of Wenzhou Medical University, Wenzhou, China; ^3^ Engineering Research Center of Clinical Functional Materials and Diagnosis & Treatment Devices of Zhejiang Province, Wenzhou Institute, University of Chinese Academy of Sciences; Oujiang Laboratory (Zhejiang Lab for Regenerative Medicine, Vision and Brain Health), Wenzhou, China

**Keywords:** *Escherichia coli*, klebsiella pneumoniae, quercetin, synergy mechanism, colistinresistance

## Abstract

Colistin is being considered as “the last ditch” treatment in many infections caused by Gram-negative stains. However, colistin is becoming increasingly invalid in treating patients who are infected with colistin-resistant *Escherichia coli* (*E. coli*) and *Klebsiella Pneumoniae* (*K. pneumoniae*). To cope with the continuous emergence of colistin resistance, the development of new drugs and therapies is highly imminent. Herein, in this work, we surprisingly found that the combination of quercetin with colistin could efficiently and synergistically eradicate the colistin-resistant *E. coli* and *K. pneumoniae*, as confirmed by the synergy checkboard and time-kill assay. Mechanismly, the treatment of quercetin combined with colistin could significantly downregulate the expression of *mcr-1* and *mgrB* that are responsible for colistin-resistance, synergistically enhancing the bacterial cell membrane damage efficacy of colistin. The colistin/quercetin combination was notably efficient in eradicating the colistin-resistant *E. coli* and *K. pneumoniae* both *in vitro* and *in vivo*. Therefore, our results may provide an efficient alternative pathway against colistin-resistant *E. coli* and *K. pneumoniae* infections.

## Introduction

Antibiotics undertake a significant duty in terms of decreasing the morbidity and mortality associated with bacteria-induced infections. Nevertheless, the overuse and abuse of antibiotics have brought out the emergence of resistant bacteria (Arias and Murray, 2012). Besides, the development of novel antibiotics is considerably slow and challenging, which increased the therapeutic difficulty for infections caused by drug-resistant bacterial infections (Cordoba et al., 2015). To address this issue, new antibacterial materials and the combination of non-antibacterial and antibiotics have been regarded as a new treatment strategy in overcoming the drug resistance of bacteria (Li et al., 2020; Su et al., 2020).

As a cationic antimicrobial peptide, colistin exploits the antibacterial effect through interplaying with the lipid A section of lipopolysaccharide (LPS) and destroys the Gram-negative bacteria outer membrane afterward (Phan et al., 2017; Shen et al., 2018; Tran et al., 2018). Despite colistin remaining high-efficient in antimicrobial activity, the massive use of colistin as a clinical therapeutic and forage additive has led to the emergence of colistin resistance (Liu et al., 2016; Xavier et al., 2016). Since colistin has been regarded as ‘the last ditch’ treatment for the infections caused by Gram-negative pathogens, it is highly desired to develop novel strategies to overcome the colistin resistance in bacteria or to resensitize the bacteria to colistin treatment.

Quercetin is a flavanol compound which is widely existed in the plant kingdom and has a variety of biological activities such as antibacterial and anti-inflammatory efficiency (Vilaca et al., 2012). Previous studies have shown that the combination of quercetin with either tetracycline or meropenem can destroy the cellular integrity and downregulate the expression of the drug-resistant genes in *E. coli* (Pal and Tripathi, 2019; Qu et al., 2019). Meanwhile, similar results were found in the combination of quercetin with other antimicrobials such as amikacin, tobramycin, levofloxacin, ceftriaxone, and gentamycin (Pal and Tripathi, 2020; Vipin et al., 2020). However, whether quercetin is efficient in overcoming the colistin resistance in bacteria, especially clinical isolates, remains undiscovered.

Herein, the synergism of the colistin/quercetin combination in eradicating the 12 colistin-resistant *E. coli* and *K. pneumoniae* clinical isolates was studied. The antimicrobial efficiency of the colistin/quercetin combination was studied both *in vitro* and *in vivo*. Also, the expression of the genes related to drug resistance is determined in bacteria after the colistin/quercetin treatment to elucidate the mechanism of quercetin in alleviating the colistin resistance in the tested clinical isolates.

## Materials and Methods

### Bacterial Strains and Chemicals

The twelve non-duplicated colistin-resistant *E. coli* and *K. pneumoniae* clinical isolates (see Table 1) were recovered from the First Affiliated Hospital of Wenzhou Medical University from 2016 to 2018. All isolates were identified by matrix-assisted laser desorption/ionization time-of-flight mass spectrometry (MALDI-TOF/MS; bioMérieux, Lyons, France). Tested strains were frozen in Luria Bertani (LB) broth medium (Sigma-Aldrich, Saint Louis, United States) containing 30% glycerol at -80°C before use.

**TABLE 1:** The MIC values against the 12 clinical isolates.

Species	Strains^a^	MIC values^b^ (μg/ml)									
		ATM	CAZ	FEP	IPM	CIP	LVX	GEN	TOB	COL	Que
*E. coli*	**DC90**	≥32^R^	≥32^R^	≥32^R^	≥8^R^	≥2^R^	≥4^R^	≥16^R^	≥16^R^	≥4^R^	≥32
	**DC3411**	≥32^R^	≥32^R^	≥32^R^	≤2	≥2^R^	≥4^R^	≤4	≤4	≥4^R^	≥32
	**DC3539**	≥32^R^	≥32^R^	≥32^R^	≤2	≥2^R^	≥4^R^	≥16^R^	≥16^R^	≥4^R^	≥32
	**DC3737**	≥32^R^	≥32^R^	≥32^R^	≤2	≥2^R^	≥4^R^	≥16^R^	≤4	≥4^R^	≥32
	**DC3806**	≥32^R^	≥32^R^	≥32^R^	≤2	≥2^R^	≥4^R^	≥16^R^	≥16^R^	≥4^R^	≥32
	**DC3846**	≥32^R^	≥32^R^	≥32^R^	≤2	≥2^R^	≥4^R^	≥16^R^	≥16^R^	≥4^R^	≥32
*K. pneumoniae*	**FK610**	≥32^R^	≥32^R^	≥32^R^	≥8^R^	≥2^R^	≥4^R^	≥16^R^	≥16^R^	≥4^R^	≥32
	**FK1913**	≥32^R^	≥32^R^	≥32^R^	≤2	≤0.5	≤1	≤4	≤4	≥4^R^	≥32
	FK2066	≤8	≤8	≤8	≤2	≤0.5	≤1	≤4	≤4	≥4^R^	≥32
	**FK4134**	≤8	≥32^R^	≥32^R^	≤2	≥2^R^	≥4^R^	≥16^R^	≥16^R^	≥4^R^	≥32
	**FK6663**	≥32^R^	≥32^R^	≥32^R^	≥8^R^	≥2^R^	≥4^R^	≥16^R^	≥16^R^	≥4^R^	≥32
	**FK6696**	≥32^R^	≥32^R^	≥32^R^	≥8^R^	≥2^R^	≥4^R^	≥16^R^	≥16^R^	≥4^R^	≥32

a Strain numbers in bold indicate they are multi-drug resistant.

b Values in bold indicate bacterial resistance.

GNB, Gram-negative bacteria; ATM, aztreonam; CAZ, ceftazidime; FEP, cefepime; IMP, imipenem; CIP, ciprofloxacin; LVX, levofloxacin; GEN, gentamicin; TOB, tobramycin; COL, colistin; Que, Quercetin.

Quercetin was purchased from MedChem Express (MCE) Co., Ltd. (New Jersey, United States), and all antibiotics used in this study, including colistin, aztreonam, ceftazidime, cefepime, imipenem, ciprofloxacin, levofloxacin, gentamicin, tobramycin, and amikacin were purchased from Wenzhou Kangtai Biological Technology Co., Ltd. (Zhejiang, China).

### Determination of Antimicrobial Resistance Profiles

Minimum inhibitory concentrations (MICs) of commonly used antibiotics, colistin, and quercetin were judged by the broth microdilution in cation-adjusted Mueller-Hinton Broth (CAMHB) (Thermo Fisher Scientific, America). The breakpoint scoring was interpreted according to the Clinical and Laboratory Standards Institute (CLSI, 2020) for both *E. coli* and *K. pneumoniae*. Briefly, in the 96-well plates, bacterial suspension (5 × 10^5^ CFU/ml) was mixed with CAMHB medium containing drugs with concentrations ranging from 0.016–32 µg/ml. The plates were incubated at 37°C overnight. The MIC was defined as the lowest drug concentration that completely inhibited the development of the microorganism, observed by the naked eye. The breakpoint of antibiotics was interpreted accordingly to the CLSI. Each MIC test against all isolates was performed in duplicate and repeated three times.

### Synergism Testing

The checkerboard assay was conducted to explore the synergy effect between colistin and quercetin *via* determining the fractional inhibitory concentration (FIC) index (Yu et al., 2020). Briefly, colistin was continuously dilute 2-fold along the *x*-axis, while quercetin was continuously diluted 2-fold along the *y*-axis to establish a matrix in 96-well plates, where each well contained different concentrations of two drugs. The bacterial suspension (100 μL) was added to each well to reach an ultimate bacterial concentration of approximately 5 × 10^5^ colony-forming unit (CFU)/mL. Bacteria in the absence of antibiotic exposure served as the control. MIC was taken as the lowest concentration of antibiotics that can inhibit the growth of bacteria.

The combination inhibition effect for each strain was confirmed by through the FIC index: FIC_colistin_ = MIC_colistin_ in combination/MIC_colistin_ alone; FIC_quercetin_ = MIC_quercetin_ in combination/MIC_quercetin_ alone; FIC index = FIC_colistin_ + FIC_quercetin_. Synergy judgment criteria were: FICI ≤0.5 indicated synergism; 0.5 < FICI≤1 indicated additive effect; 1 < FICI≤2 indicated irrelevant effect; FICI >2 indicated antagonistic effect (Qu et al., 2019).

### Time-Kill Assay

The time-kill assay was carried out according to a published experimental process (Berard et al., 2016) with minor modifications. Briefly, colistin-resistant *E. coli* (*n* = 6) or *K. pneumoniae* (*n* = 6) (1 × 10^6^ CFU/ml) were inoculated in 10 ml CAMHB containing colistin (½ MIC), quercetin (½ MIC), or quercetin (½ MIC)+colistin (½ MIC). Bacteria in the CAMHB medium without antibiotics treatment served as the control. The tubes were incubated at 37°C. At various time points (0, 2, 4, 6, 12, and 24 h) post-incubation, an aliquot (100 µL) of bacterial suspension was withdrawn and spotted on Mueller-Hinton agar plates, incubated overnight at 37°C, followed by CFU counting (Zhang et al., 2019). A decrease ≥3 log10 for CFU/mL by 24 h was defined as the bactericidal activity, and a decrease ≥2 log10 for CFU/mL was defined as synergistic activity calculated by comparison of two drugs combination and single drug treatment (Berard et al., 2016).

### Quantitative Real-Time PCR (qRT-PCR)

Quantification of expression of *mcr-1*and *mgrB* genes in *E. coli* and *K. pneumoniae* respectively were performed using reverse transcription PCR (qRT-PCR) (Jayol et al., 2014; Gaudereto et al., 2019). *16SrRNA* and *rpoB* served as standardized reference genes for *E. coli* and *K. pneumoniae*, respectively (Tadesse et al., 2012; Liu et al., 2017). Primers were composited by Shanghai Huada Biological Engineering Co., Ltd (Shenzhen, China). A commercial RNA extraction kit was used to isolate total RNA from the strains cultured in LB broth containing quercetin (½ MIC), colistin (½ MIC), or quercetin (½ MIC)+colistin (½ MIC) for 16–18 h (Tiangen Biotech, Beijing, China) according to the user’s guideline (Bulman et al., 2017). Quantified gene expression by the ∆∆Ct method. Experiments for each gene were performed in triplicate.

### Bacterial Cell Permeability

To study the damage of various antibiotics treatments on the bacterial cell membrane, *E. coli* and *K. pneumoniae* (OD_600_ = 0.5) were inoculated into LB broth containing quercetin (½ MIC), colistin (½ MIC), and quercetin (½ MIC)+colistin (½ MIC) (Qu et al., 2019), followed by incubation in a shaker (180 rpm) at 37°C for 24 h. Then, these suspensions were centrifuged at 5,000 rpm for 5 min. The supernatant was collected and subjected to the alkaline phosphatase (ALP) activity test *via* a corresponding kit (Solarbio, Beijing). All experiments were performed in triplicate. In general, AKP/ALP uses *p*-nitrophenyl phosphate (pNPP) as a phosphatase substrate which turns yellow (*λ*_max_ = 405 nm) when dephosphorylated by ALP.

Similarly, the *β*-galactosidase activity in the supernatant was tested via its corresponding kit (Solarbio, Beijing). *β*-galactosidase decomposes *p*-nitrobenzene-*β*-d-galactopyranoside into *p*-nitrophenol, and the activity of *β*-galactosidase is calculated by measuring its absorbance at 420 nm.

### Scanning Electron Microscope (SEM)

To study the morphological changes of the bacterial cell membrane after various treatments, SEM images of bacteria were taken (Watanabe et al., 2018). Briefly, a glass slide (24 × 24 mm in size) was plated in each well of a six-well plate, then, bacteria (1.5 × 10^8^ CFU/ml) in 2 ml MH broth containing quercetin (½ MIC), colistin (½ MIC), or quercetin (½ MIC)+colistin (½ MIC) were added to each well. The plate was incubated at 37°C for 18 h. Subsequently, the glass slides were taken out, washed three times with PBS. The remaining bacteria were fixed in 500 µL 5% (v/v) glutaraldehyde (Solarbio, Beijing) and incubated overnight at 4°C. After washing three times in 1×PBS, the cells were treated with air drying and platinum coating, then observed by SEM (S-3000N, Japan).

### Eradication of Bacterial Infection *in vivo*

To establish the murine infection model, specific-pathogen-free (SPF) female ICR (Institute of Cancer Research) mice, 5–6 weeks old (Charles River, Hangzhou, China), were used. Mice were raised according to the National Standards for Laboratory Animals of China (GB 14925–2010). All animal experiments were approved by the Zhejiang Association for Science and Technology SYXK (ID: SYXK (Zhejiang)2018–0017) and carried out according to the guideline of the Wenzhou Laboratory Animal Welfare and Ethics.

In short, mice were intraperitoneally injected with cyclophosphamide (Solarbio, Beijing) at 4 days (150 mg/kg) and one day (100 mg/kg) before thigh intramuscular injection to induce a neutropenia model (≤100 neutrophils/mm^3^). Then mice were divided into four groups (3 mice per group), each posterior thigh muscle of mice was exponentially injected with 100 μL of bacterial suspension (1.5 × 10^7^ CFU/ml). At 2 h post bacterial inoculation, mice were administered with 1) 1×PBS (untreated group), 2) quercetin (50 mg/kg), 3) colistin/quercitin combination (5 mg/kg colistin +50 mg/kg quercetin, or 7.5 mg/kg colistin +50 mg/kg quercetin) by intraperitoneal injection. At 24 h post-treatment, mice were euthanized through cervical dislocation. The posterior thigh tissue was collected, homogenized, diluted in PBS, spread on agar plates, and cultured at 37°C overnight for CFU quantification.

### Statistical Analysis

With regard to all analyses, a *p* value <0.05 was defined to be statistically significant. Paired two-tailed *t*-test and one-way ANOVA were performed to compared changes in gene expression level, cell membrane, and *in vivo* bacterial colony counts. All statistically calculated values were carried out with SPSS v.22.0 software (SPSS Inc, Chicago, IL, United States).

### Ethics

The patients provided their written informed consent to participate in this study. The study and consent procedure was approved by the ethics committee of the First Affiliated Hospital of Wenzhou Medical University (No. 2020140).

## Results

### Susceptible Testing of Antimicrobial Resistance Profiles

The MIC of several commonly used antibiotics and quercetin were tested against the clinically isolated *E. coli* and *K. pneumoniae*, as well as *E. coli* ATCC 25922 (drug-susceptible control). As shown in Table 1, the MIC value of colistin was tested to be 4–32 µg/ml for the majority of the tested *E. coli* and *K. pneumoniae* strains. Of note, 1 *E. coli* strain, DC90, and 1 *K. pneumoniae* strain, FK 1913, were found extremely resistant to colistin treatment (MIC ≥64 µg/ml). These results suggest the colistin resistance of those clinically isolated *E. coli* and *K. pneumoniae* strains. Moreover, these clinical isolates were resistant to the majority of the commercially available antibiotics as exemplified in Table 1. Ten strains showed a multidrug-resistant phenotype. Specifically, all the six *E. coli* strains were tested resistant to the treatment of aztreonam or cefepime and 5 *K. pneumoniae* strains were resistant to cefepime. Imipenem still holds therapeutic efficacy to 5 *E. coli* strains and three *K. pneumoniae* strains. The MIC values of the quality control strains are all within the acceptable range.

### Synergism Test by the Checkerboard Method

The checkerboard method was applied to test the synergism between colistin and quercetin against the 12 clinical isolates. As shown in Table 2, a 4–32-fold decrease in MIC of colistin for four *E. coli* (DC90, DC3539, DC3737, and DC3846) and four *K. pneumoniae* (FK610, FK 1913, FK4134, and FK6663) was observed when colistin was used in combination with quercetin. For those strains, the FIC values were calculated to be 0141–0.375, suggesting an excellent synergism between colistin and quercetin. The quercetin/colistin combination exhibited the additive effect (FIC = 0.75) against two *E. coli* (DC3411, DC3806) and two *K. pneumoniae* (FK 2066, FK6696) strains.

**TABLE 2:** Summary of MIC values and FICI of colistin combined with quercetin against the 12 colistin-resistant *E. coli* and *K. pneumoniae* clinical isolates.

Strain	Antimicrobial susceptibility (MIC, μg/mL)		Antimicrobial combination (MIC, μg/mL)			
	CST	Que	CST + Que	FICI	Potentiation	Interpretation
DC90	64	256	2 + 32	0.4388	32-fold	synergy
DC3411	4	256	1 + 128	0.75	4-fold	additive
DC3539	4	256	1 + 32	0.375	4-fold	synergy
DC3737	8	512	2 + 64	0.375	4-fold	synergy
DC3806	4	256	1 + 32	0.75	4-fold	additive
DC3846	8	256	2 + 128	0.375	4-fold	synergy
FK610	16	256	4 + 64	0.50	4-fold	synergy
FK1913	64	256	8 + 16	0.3125	8-fold	synergy
FK2066	32	512	16 + 128	0.75	2-fold	additive
FK4134	8	256	2 + 32	0.375	4-fold	synergy
FK6663	32	256	8 + 32	0.375	4-fold	synergy
FK6696	32	256	16 + 64	0.75	2-fold	additive

### Time-Kill Assay

**FIGURE 1:**
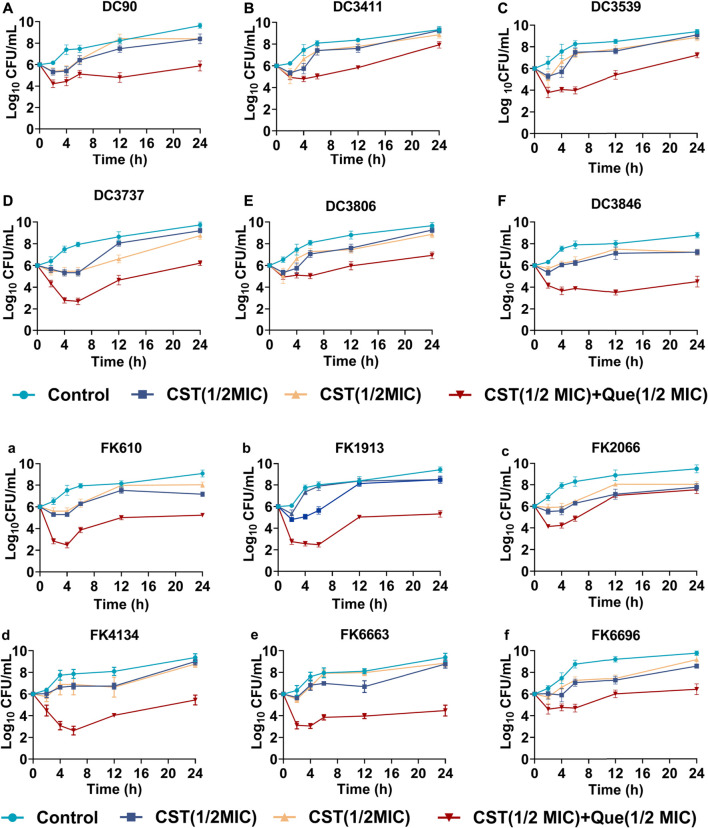
Bactericidal kinetics of various treatments against the colistin-resistant *E. coli* and *K. pneumoniae* **(A–F)** Quantified log10 CFU/mL of various colistin-resistant *E. coli* strains at different time intervals after being exposed to various treatments **(G–L)** Quantified log10 CFU/mL of various colistin-resistant *K. pneumoniae* strains at different time intervals after being exposed to various treatments.

Time–kill assay was carried out to further demonstrate the synergistic drug combination of quercetin and colistin against the clinical isolates. As shown in Figure 1, in the absence of antibiotic or quercetin exposure (control group), bacteria grew rampantly in a conventional gradient growth trend. The treatment of either colistin or quercetin at a concentration of 1/2 of its corresponding MIC slightly inhibited the bacterial growth within the first 6 h, after which the bacterial growth was negligibly affected, and there is no notable difference between the drug-treated groups and the control group after 24-h incubation. Of note, the colistin/quercetin combination was efficient in eradicating those clinical isolates. The majority of the bacteria were killed (a reduction of 2–4 in log10 CFU/mL) by the colistin/quercetin combination within the first 6 h, after which the bacterial growth was still largely inhibited. As a result, a reduction of 2-4 in log10 CFU/mL was observed in the bacterial suspension treated with the colistin/quercetin combination compared with the bacteria in the control group, regardless of the bacterial strains.

### Gene Expression

**FIGURE 2:**
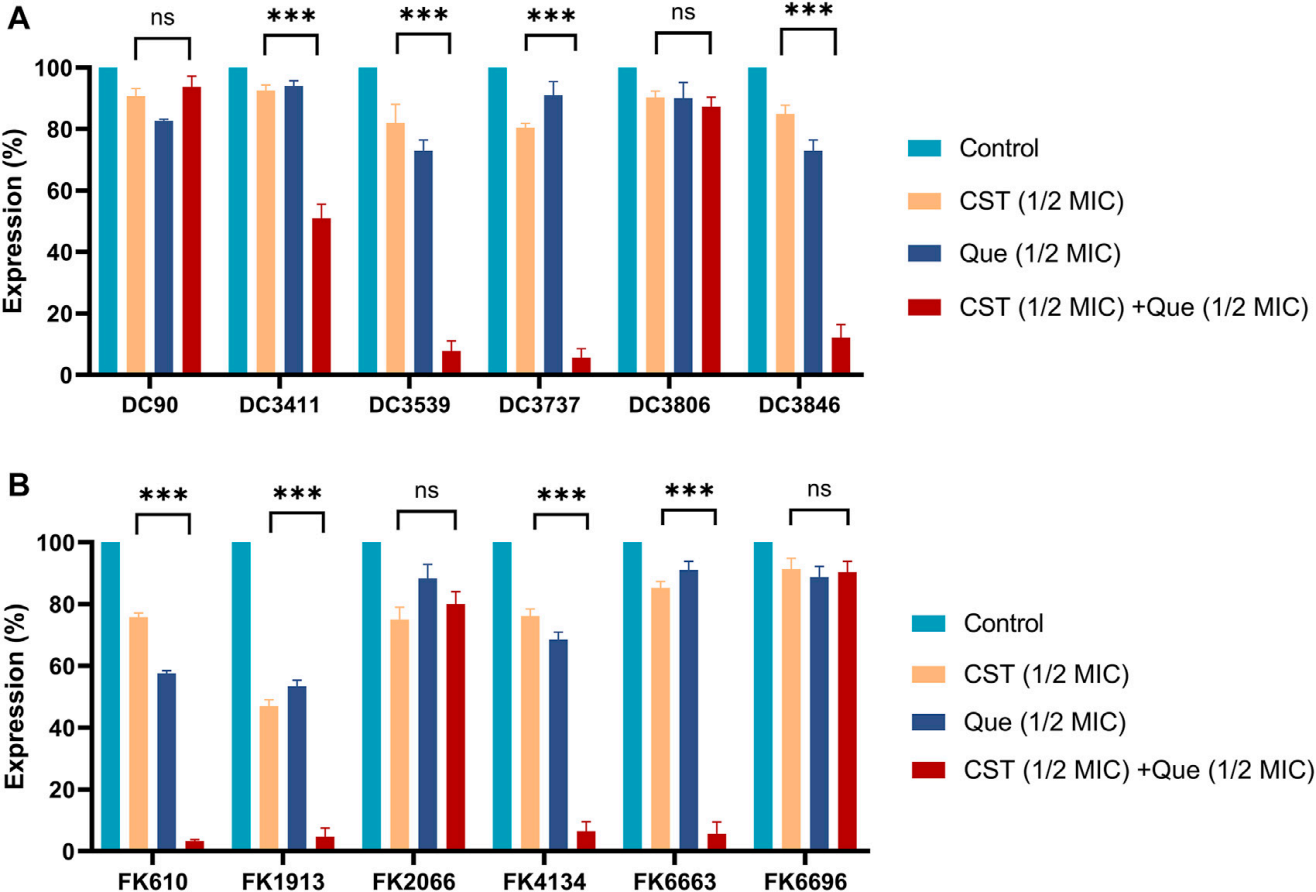
Combination of colistin with quercetin significantly downregulates the mRNA levels of *mcr-1* and *mgrB* genes in *E. coli* and *K. pneumoniae*, respectively **(A)** Relative mRNA levels of *mcr-1* in different *E. coli* strains after the various treatments **(B)** Relative mRNA levels of *mgrB* in different *K. pneumoniae* strains after the various treatments. *16srRNA* and *rpoB* were used as the internal reference for *E. coli* and *K. pneumoniae*, respectively, and the mRNA levels of those two genes were taken as 100%. All experiments were performed in triplicate. The results were displayed as means ± standard deviations over the three independent experiments. ns stands for not significant, *p < 0.05, **p < 0.01, ***p < 0.001, determined by the Student’s *t*-test.

To figure out the detailed mechanism of the colistin/quercetin combination, the gene expression of *mcr-1* in *E. coli* and *mgrB* in *K. pneumoniae* after various treatments was studied using qRT-PCR. As illustrated in Figure 2, the treatment of either colistin or quercetin alone could inhibit up to 12% of *mcr-1* expression in *E. coli* and up to 50% of *mgrB* expression in *K. pneumoniae.* The colistin/quercetin combination exhibited notably higher efficiency in suppressing the expression of *mcr-1* and *mgrB*, a up to 90% reduction of *mcr-1* expression in *E. coli*, and up to 95% reduction of *mgrB* expression in *K. pneumoniae* was observed in the majority of the clinical isolates. Of special note, in strains such as *E. coli* DC3806 and *K. pneumoniae* FK6696, the colistin/quercetin combination did not induce the significant gene suppression efficiency compared to the monotherapy of either colistin or quercetin.

### Permeability of Bacterial Cell Membrane

**FIGURE 3:**
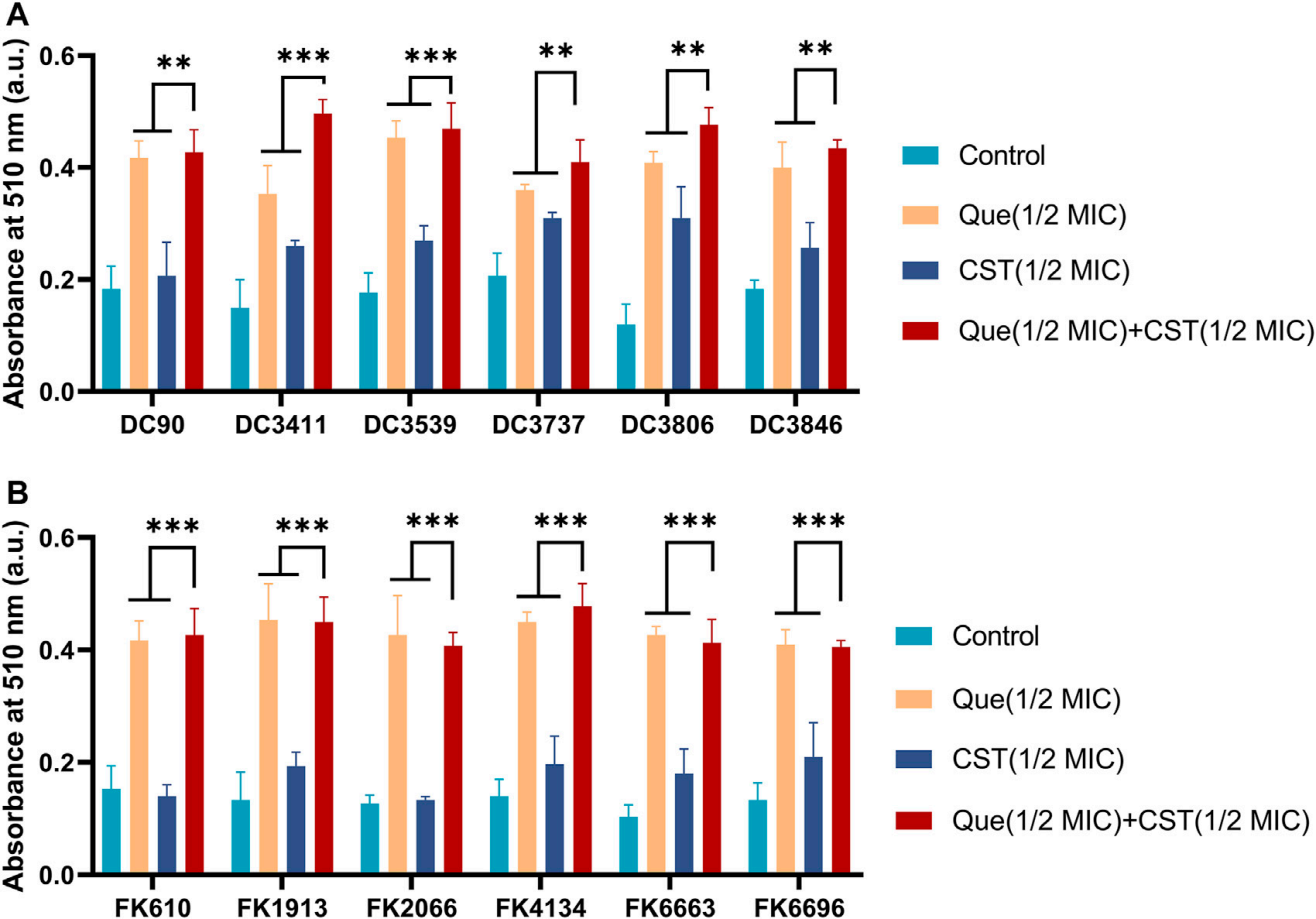
Combination of quercetin and colistin contributes to the damage of bacterial cell membranes and the leakage of bacterial ALPs. The absorbance at 510 nm integrity of *E. coli* **(A)** and *K. pneumoniae* **(B)** by detecting ALP. The chart indicates that an increase for alkaline phosphatase in the medium after exposure to the specified compound. The results are shown as the mean and standard deviation of three independent experiments. ns stands for not significant, *p < 0.05, **p < 0.01, ***p < 0.001, determined by the Student’s *t*-test.

**FIGURE 4:**
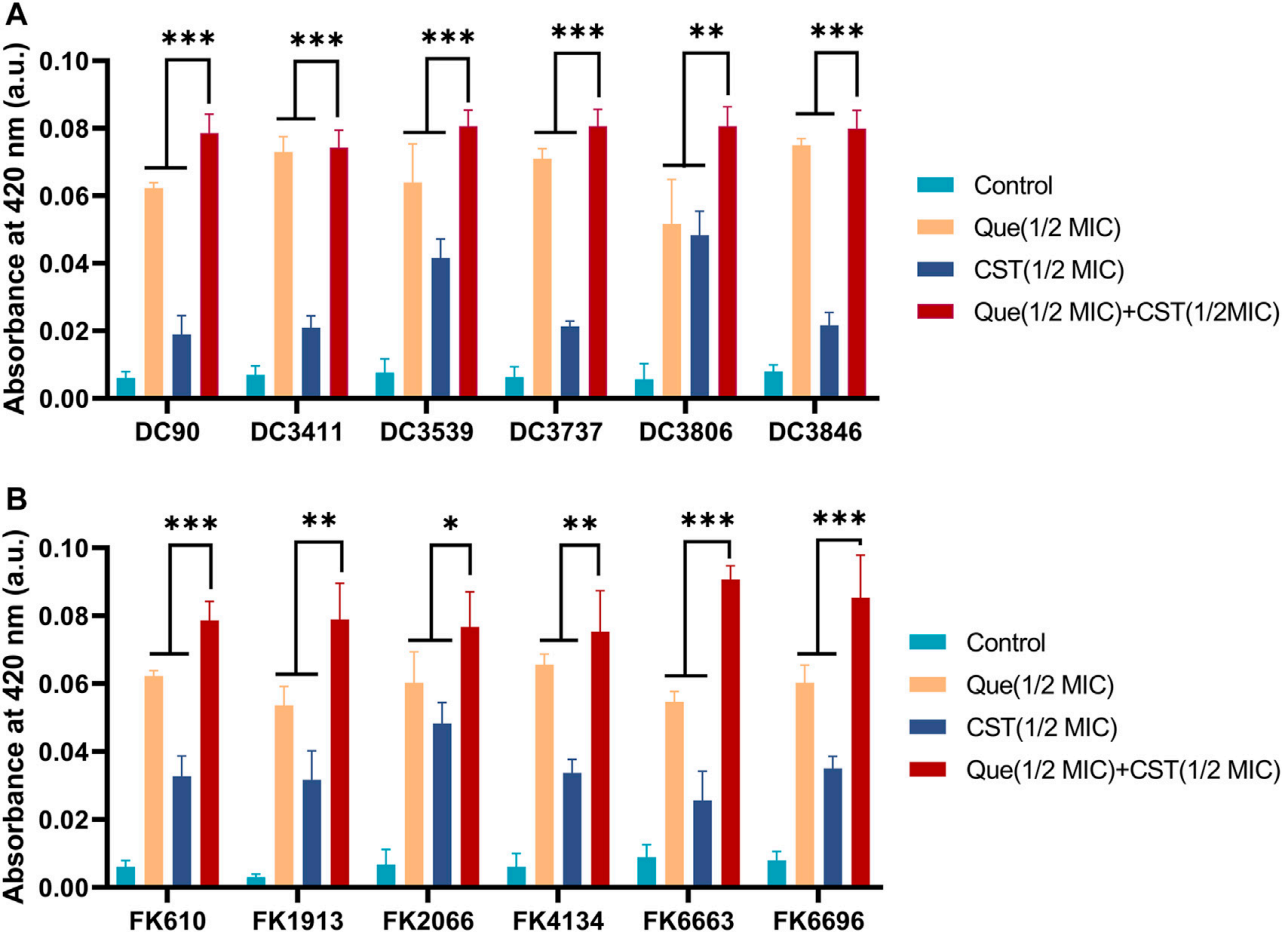
Combination of quercetin and colistin contributes to the damage of bacterial cell membranes and the leakage of bacterial β-galactosidases. Quercetin and colistin combine to disrupt bacterial cell wall integrity of *E. coli* **(A)** and *K. pneumoniae* **(B)** by detecting the release of intracellular β-galactosidase. The chart indicates that an increase for β-galactosidase in the medium after exposure to the specified compound. The results are shown as the mean and standard deviation of three independent experiments. ns stands for not significant, *p < 0.05, **p < 0.01, ***p < 0.001, determined by the Student’s *t*-test.

Subsequently, the permeability of the bacterial cell membrane after various treatments was investigated. Extracellular ALPs and *β*-galactosidases after various treatments were determined using their corresponding assays. As shown in Figures 3, 4, upon the treatment of quercetin, the amount of extracellular ALPs and *β*-galactosidases was notably enhanced in both *E. coli* and *K. pneumoniae*, suggesting the damage of the bacterial cell membrane. Although colistin also triggered a notable ALPs release from bacterial cells, its efficiency was not comparable to that of quercetin. Furthermore, the colistin/quercetin combination induced similar or better bacterial cell membrane damage efficiency compared to quercetin treatment alone in all the tested isolates.

### Scanning Electron Microscope (SEM)

Furthermore, the change of bacterial morphologies after various treatments was investigated *via* SEM. The colistin/quercetin combination induced significant bacterial cell membrane damage. Whereas the cell morphology of bacteria after the treatment of quercetin or colistin at its corresponding FIC concentration was similar to that of the PBS-treated bacteria.

### *In vivo* Bactericidal Efficacy Elevation

Since we have demonstrated that colistin combined with quercetin has a synergistic bactericidal effect *in vitro*, it is still important to verify the effectiveness of the combination through *in vivo* experiments, especially animal models. Herein, a murine model with *E. coli* and *K. pneumoniae* infection was established and received various treatments. As shown in Figure 6, monotherapy treatment with quercetin at a dose of 50 mg/kg only slightly inhibited the growth of *E. coli* DC1913 and *K. pneumoniae* FK3737, as illustrated by a reduction of 1.01 and 0.16 in log_10_ CFU/thigh in *E. coli* DC1913 and *K. pneumoniae* FK3737, respectively, compared with the PBS-treated mice. The treatment of colistin at a dose of 5 mg/kg every 24 h only slightly inhibited the bacterial growth with a reduction of 0.08–0.51 in log_10_ CFU/thigh in *E. coli* DC 1913- or *K. pneumoniae* FK3737-infected mice. However, a higher dose of 7.5 mg/kg could efficiently enhance the therapeutic performance of colistin, yielding an ∼1.06 reduction in log_10_ CFU/thigh in the infected mice. Particularly, the combination of quercetin (50 mg/kg) and colistin (5 mg/kg) achieved a log10 reduction of 1.522 for *E. coli* DC1913 and 1.438 for *K. pneumoniae* FK3737, respectively, compared to the PBS-treated mice. Moreover, dose-dependent bacterial eradication efficacy was observed, as illustrated by a log10 reduction of 2.304 for *E. coli* DC1913 and 2.017 for *K. pneumoniae* FK3737 was achieved when the infected mice were treated with quercetin (50 mg/kg) and colistin (7.5 mg/kg). These results collectively suggest the colistin/quercetin combination is efficient in eradicating the colistin-resistant *E. coli* and *K. pneumoniae* infections *in vivo*, posing a great potential in clinical translation.

## Discussion

Colistin has been considered as the “last-line” therapy against infections caused by multidrug-resistant Gram-negative pathogens (Shen et al., 2020). Previous studies and clinical practice have shown that colistin may cause nephrotoxicity and neurotoxicity (Gregoire et al., 2017). Therefore, WHO has listed colistin as one of the critically vital antibiotics that need to be replaced (Biswas et al., 2014). As a result, the clinical use of colistin was discontinued and replaced with other effective antibiotics. However, the increasing challenges caused by multi-drug resistant Gram-negative bacteria, together with the lack of new type antibiotics against such strains have made colistin come back into the spotlight (Liu et al., 2021; Su et al., 2021). Worse still, many clinically isolated pathogens are resistant to colistin treatment. Therefore, there is a dire need to develop new techniques to overcome the drug resistance of bacteria against colistin or to make the resistant bacteria resensitize to colistin treatment again. In this research, we developed a novel strategy to rejuvenate the power of colistin against the colistin-resistant clinical isolates *E. coli* and *K. pneumoniae via* the combination of colistin and quercetin.

Quercetin is a flavonol compound that widely exists in edible plants with low toxicity to humans and animals and can be used for therapeutic purposes as an eatables supplement (3 times a day, 250–500 mg each time). Of note, quercetin can scavenge free radicals to prevent lipid peroxidation, inhibiting tumor proliferation (Reyes-Farias and Carrasco-Pozo, 2019). Also, it has been reported that the combination of quercetin with other antibiotics such as vancomycin and tetracycline possess synergistic antibacterial activity against *E. coli* and *Staphylococcus aureus* (Qu et al., 2019; Yu et al., 2020). In addition, quercetin possesses low cytotoxicity and can increase intracellular oxidative stress in tumor cells usually at concentrations >40 mM(Vargas and Burd, 2010). However, the effect of quercetin on the antimicrobial efficacy of colistin has never been shown.

In this work, we presented the first report of the synergistic activity between quercetin and colistin against the colistin-resistant *E. coli* and *K. pneumoniae* clinical isolates. The MIC of colistin was reduced by 2–32-fold when it combined with quercetin, suggesting that quercetin and colistin combination therapy could be a promising treatment option to confront the infections caused by colistin-resistant *E. coli* and *K. pneumoniae*. We also carried out the time-killing assays to evaluate *in vitro* antibacterial activity of the quercetin/colistin combination. We confirmed that the quercetin/colistin combination is efficient to eradicate the colistin-resistant *E. coli* and *K. pneumoniae.* Besides, the previous study has found out that the expression of *mcr-1* and *mgrB* genes in colistin-resistant *E. coli* and *K. pneumoniae* are upregulated (Liao et al., 2020). *mcr-1* is a plasmid-mediated *E. coli* resistance gene, which is widely spread in the world to cause colistin resistance (Jiang et al., 2020). When it comes to *K. pneumoniae, mgrB* gene is considered to be a common cause of colistin resistance. After using the combination of quercetin and colistin, we noticed that the mRNA levels of *mcr-1* and *mgrB* genes were significantly decreased in most strains (Figure 2). Of note, the genes of interest were not down-regulated in several strains, probably due to the differences between strains and the complexity of colistin resistance mechanism, which could be a promising topic for our future studies.

**FIGURE 5:**
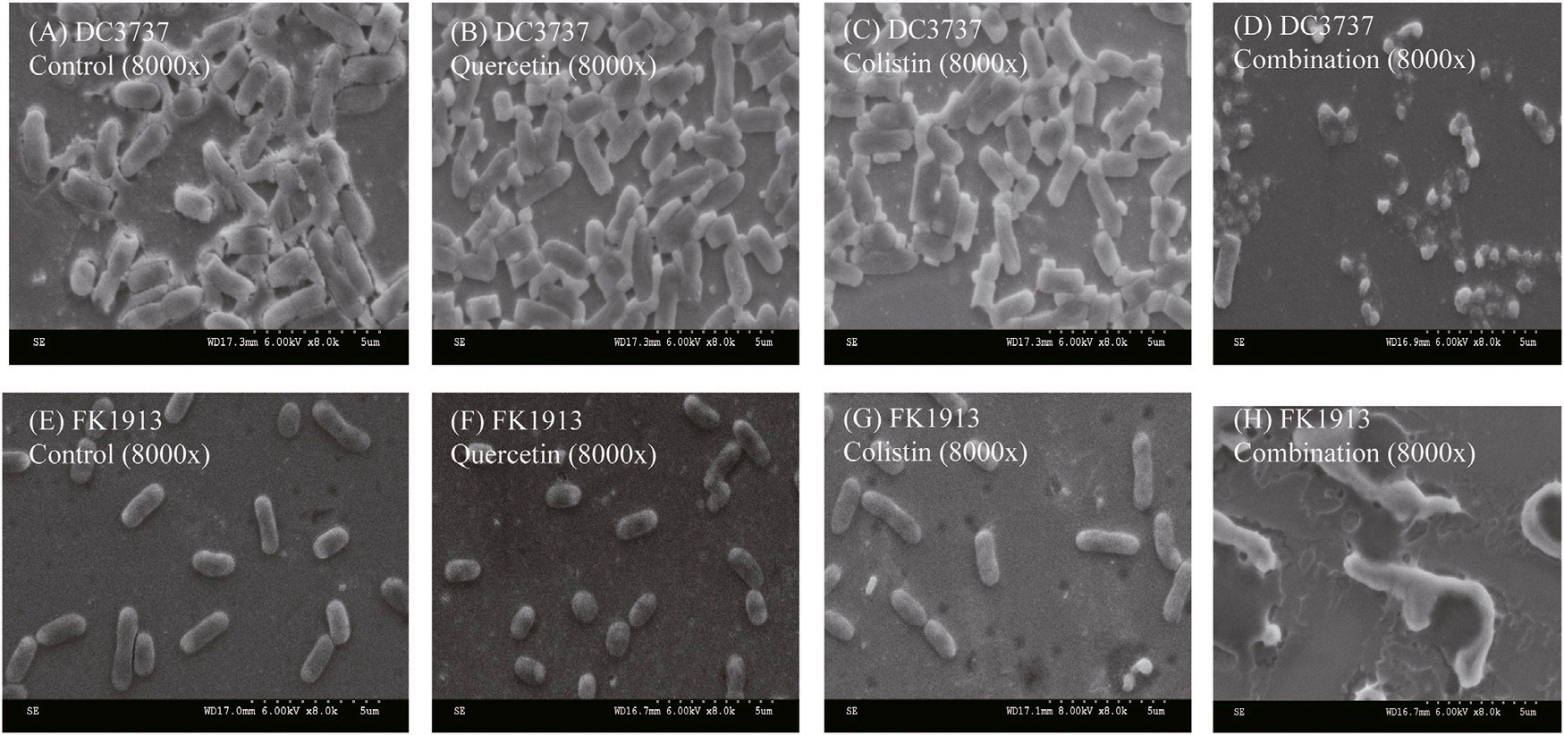
SEM images showing the morphological change of bacterial cell membrane of *E. coli* DC3737 and *K. pneumoniae* FK1913 after various treatments.

Similar to many other natural extracts, quercetin can exert antibacterial activity to a certain extent (Jaisinghani, 2017; Hao et al., 2021; Zhu et al., 2021). Although these natural extracts may differ in their antimicrobial mechanism, most of them induce bacterial lysis *via* cell membrane damage. For example, baicalein, another flavonoid, can disturb cell wall integrity by binding peptidoglycan directly (Zhao et al., 2001). This phenomenon was also observed in our experiment where intercellular bacterial ALPs and *β*-galactosidases were released after the treatment of quercetin/colistin combination (Figures 3, 4 **)**. Furthermore, we observed the morphological change of bacterial envelopes after the treatment of quercetin/colistin combination using a scanning electron microscope (Figure 5). Taken together, these results indicated that the combination therapy could efficiently destroy the integrity of the bacterial cell membrane.

**FIGURE 6:**
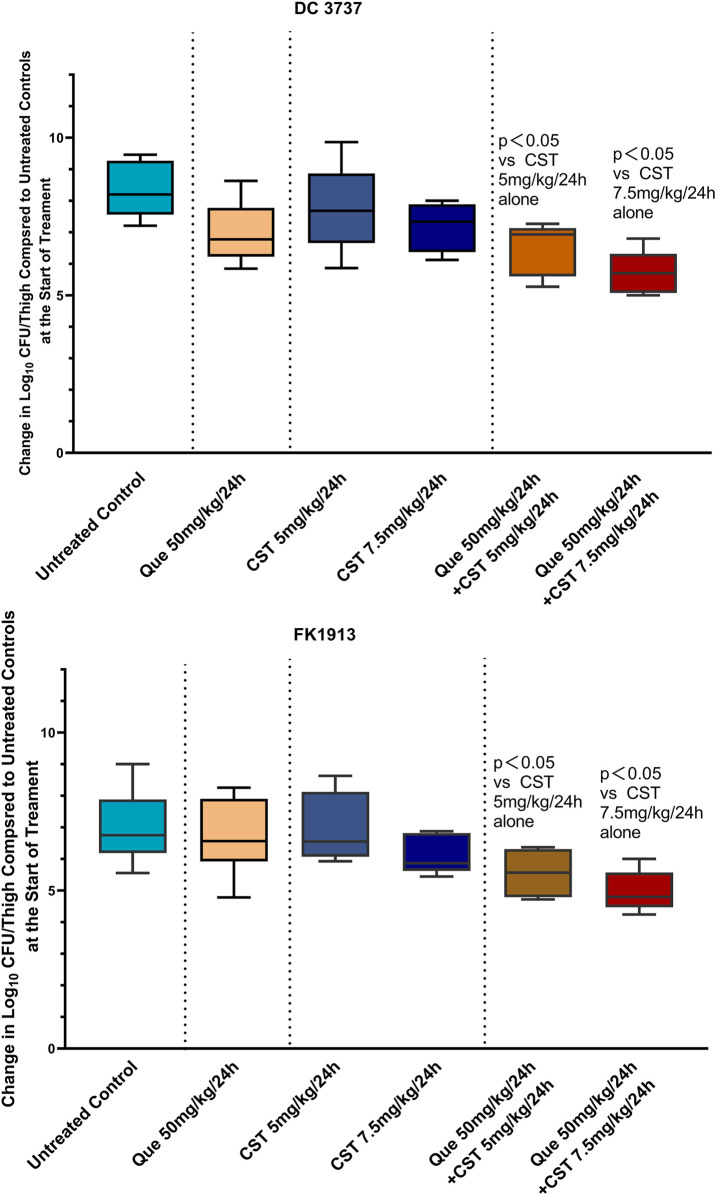
Quantified log10 CFU/thigh in mice at 24 h post-treatment of various treatments. Changes in mouse thigh muscles (Δlog_10_ cfu/thigh) after 24 h of monotherapy or combination therapy using different dosing regimens against two colistin-resistant strains of *E. coli* (DC3737) and *K. pneumoniae* (FK 1913) (n = 6).

Encouraged by the excellent synergism of quercetin/colistin combination, we further constructed the murine infection model and evaluated the antimicrobial efficacy of quercetin/colistin combination *in vivo.* Notably, colistin combined with quercetin showed that the efficacy is higher than monotherapy treatment, with 1.8–3.2 log10 CFU reduction after 24 h (Figures 6A,B), showing the significant antibacterial effect *in vivo* against *mcr-1* producing *E. coli* and *mgrB* producing *K. pneumoniae* colistin-resistant isolates. This provides a practical foundation for clinical therapy. Considering that experimental conditions cannot be completely simulated *in vivo*, such as dynamic antibiotic variety, bacterial virulence, and host immune response against infection (Zhou et al., 2017), more efforts should be made in the future to elucidate the above-mentioned mechanisms or issues.

**FIGURE 7:**
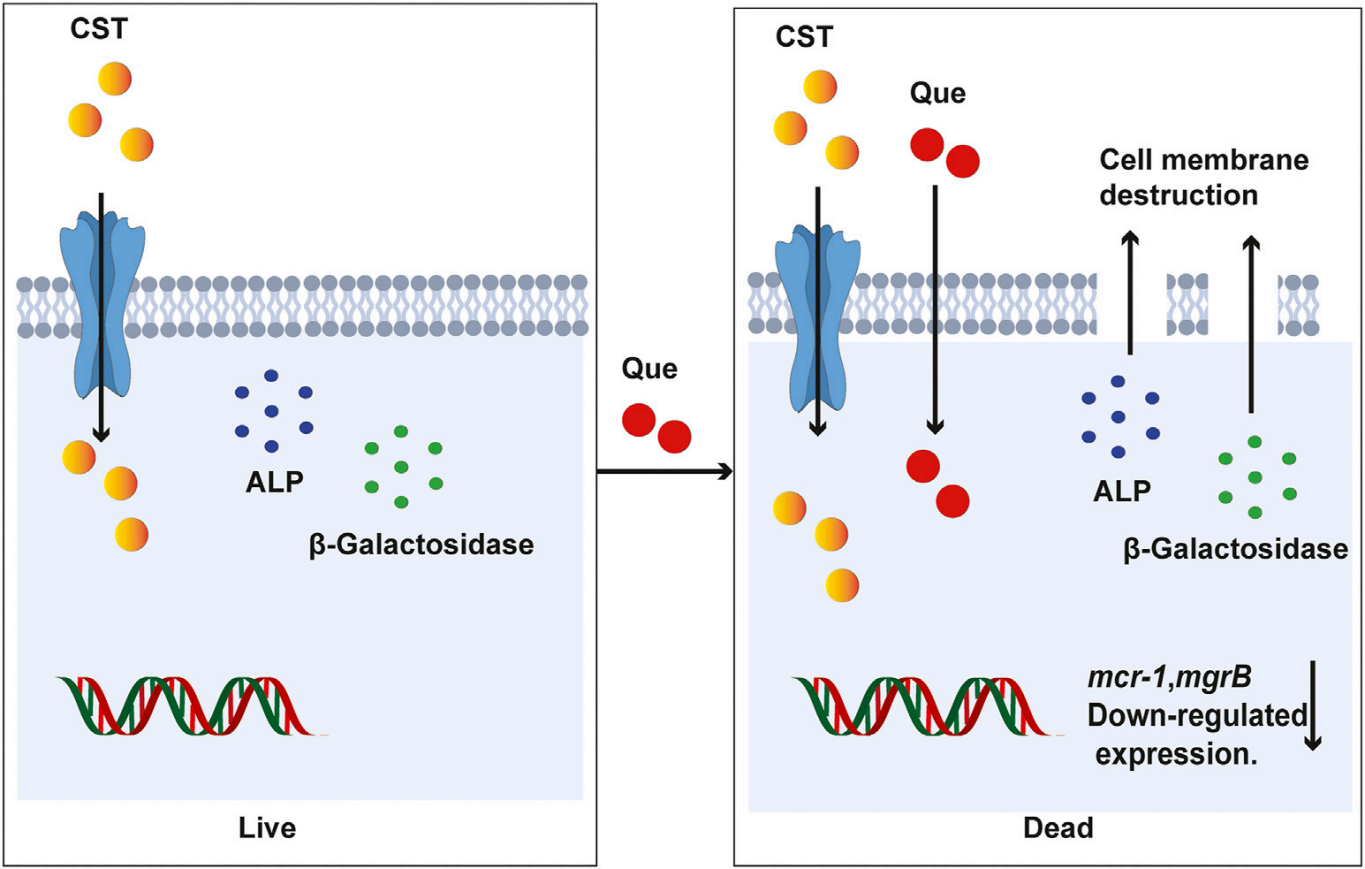
A proposed mechanism of quercetin/colistin combination in eradicating colistin-resistant *E. coli* and *K. pneumoniae*.

In summary, the combination of quercetin and colistin provides a new treatment choice for the infections caused by colistin-resistant bacteria by reducing the dosage and concentration of colistin. Quercetin could significantly resensitize the colistin-resistant *E. coli* and *K. pneumoniae* clinical isolates to colistin *in vitro*. In addition, results indicate that quercetin combined with colistin act synergistically to damage the cell membrane and wall permeability and induce an effective reduction in the expression of *mcr-1* and *mgrB* genes (Figure 7). In the murine infection model caused by the colistin-resistant *E. coli* and *K. pneumoniae* clinical isolates, the quercetin/colistin combination also showed a great synergy effect in eradicating the infected bacteria. Taken together, our work is of great significance for the clinical treatment of refractory colistin-resistant bacteria infections via teaching an old dog a new trick.

## Data Availability

The original contributions presented in the study are included in the article/Supplementary Material, further inquiries can be directed to the corresponding authors.
